# Intratemporal facial nerve ultrastructure in patients with idiopathic facial paralysis. Viral infection evidence study

**DOI:** 10.1590/S1808-86942010000500017

**Published:** 2015-10-22

**Authors:** Rosangela Aló Maluza Florez, Raquel Lang, Adriano Mora Veridiano, Renato de Oliveira Zanini, Pedro Luiz Calió, Ricardo dos Santos Simões, José Ricardo Gurgel Testa

**Affiliations:** 1 MSc in Otolaryngology - Federal University of São Paulo (UNIFESP), Full Professor - Universidade Santa Cecília (UNISANTA) Coordinator of the Dentistry School of UNISANTA; 2 DDS - UNISANTA, Professor at the Dentistry School - UNISANTA; 3 MSc in morphology - UNIFESP, Assistant Professor - Dentistry School - UNISANTA; 4 DDS - UNISANTA, Professor at the Dentistry School - UNISANTA; 5 PhD in Histology and Structural Biology - UNIFESP, Full Professor of Dentistry - UNISANTA; 6 OBGYN (TEGO), MSc Student - University of São Paulo; 7 PhD in Otolaryngology - UNIFESP, Adjunct Professor of ENT - UNIFESP. Departamento de Otorrinolaringologia e Cirurgia de Cabeça e Pescoço Universidade Federal de São Paulo (UNIFESP/EPM)

**Keywords:** facial paralysis, facial nerve, facial nerve diseases, bell palsy.

## Abstract

**Abstract:**

The etiology of idiopathic peripheral facial palsy (IPFP) is still uncertain; however, some authors suggest the possibility of a viral infection.

**Aim:**

to analyze the ultrastructure of the facial nerve seeking viral evidences that might provide etiological data.

**Material and Methods:**

We studied 20 patients with peripheral facial palsy (PFP), with moderate to severe FP, of both genders, between 18-60 years of age, from the Clinic of Facial Nerve Disorders. The patients were broken down into two groups - Study: eleven patients with IPFP and Control: nine patients with trauma or tumor-related PFP. The fragments were obtained from the facial nerve sheath or from fragments of its stumps - which would be discarded or sent to pathology exam during the facial nerve repair surgery. The removed tissue was fixed in 2% glutaraldehyde, and studied under Electronic Transmission Microscopy.

**Results:**

In the study group we observed an intense repair cellular activity by increased collagen fibers, fibroblasts containing developed organelles, free of viral particles. In the control group this repair activity was not evident, but no viral particles were observed.

**Conclusion:**

There were no viral particles, and there were evidences of intense activity of repair or viral infection.

## INTRODUCTION

Peripheral facial paralysis (PFP) causes not only physical deformities, but also functional and often times psychological problems, which can cause social and professional impairment to the patients. The most frequent cause of PFP is idiopathic, also known as Bell 's palsy, and the other etiologies are trauma, tumors, infections, neurologic, congenital and iatrogenic^1^, ^2^, ^3^, ^4^, ^5^, ^6^.

In PFP treatment, we are still in doubt about the efficacy of the current clinical and/or surgical measures, since the natural evolution of the disease is, usually benign.^4^,^5^ A literature review of the last century on the treatment of idiopathic PFP reveals that, since its etiology is not fully understood, treatment options are controversial and vary widely^7^, ^8^, ^9^. The goal for future investigations must be to establish the cause of facial paralysis from each patient, so that treatment can be based on accurate etiopathology information, thus increasing its efficacy.

One of the etiologies considered for Bell's PFP would be that of a facial viral neuritis, recently more specifically associated with Herpes simplex virus^10^,^11^. Although experimentally we can reproduce a setting matching that of Bell's PFP through the inoculation of viruSES in animals, it is still not yet proven in humans by the uniform depiction of viral particles in pathology studies or the finding of viable viruses in neural samples.

This study aimed at assessing the ultrastructure of the facial nerve from patients with idiopathic PFP through fragments of the nerve sheath, biopsy of its stumps or branches, looking for evidence of viral infection.

## METHODS

The present study was initially approved by the Ethics in Research Committee of the institution where it was carried out, under protocol # 11/2005. All the patients in this study agreed to participate in it and signed an Informed Consent Form.

We had 20 patients with PFP participating in this study, coming from the Facial Nerve Disorders' Ward, which were broken down into two groups: Study Group (G-PFP.I) - made up by 11 patients with idiopathic PFP or “Bell's Palsy”, who were referred to nerve decompression surgery by nerve sheath opening; and the Control Group (G-PFP.T) - made up of 9 patients with PFP of trauma or tumor origin, and who were also operated upon.

As to the inclusion criterion for surgical indication we should mention that the patients had complete facial paralysis between 20 and 45 days of development; without response from the electrophysiological tests (unresponsive electroneurography and Hilger tests). As to the time after the paralysis in which the surgery was carried out, and to better correlate it with the ultrastructural findings in the cases of idiopathic paralysis between 20 and 45 days after its onset, and in the trauma and tumor cases, they were all cases of chronic paralysis with more than 6 months of evolution without improvements.

We studied 20 fragments of facial nerve sheaths, or of their stumps, that during repair surgery of the facial nerve would be discarded or referred to pathology studies. Fragments of the tissue removed were dipped in 2% glutaraldehyde 0.5M phosphate buffer and 7.2 pH, later fixed in 1% osmium tetroxide for one hour and dipped in an aqueous solution of 0.5% uranyl acetate for 12 hours. The material was later dehydrated and embedded and included in resin (Polylite, Reichhold Research Triangle Park, NC, USA). The tissue was then put in blocks and properly prepared and sent to the Porter Blum MT-1 ultramicrotome, thus providing semi-fine slices of approximately 0.5mm thick, dyed in methylene blue. The semi-fine slice blocks, selected upon light microscopy, yielded ultrafine slices, with thicknesses varying between 40 and 80 nm which were assembled in copper screens with 200 mesh. As contrast medium, we used uranyl acetate and lead cytrate^12^,^13^. The exam of the screens and the electronic microphotographies (MP) were done in a transmission Carl Zeiss electronic microscope, EM 900 model at 80 kV.

## RESULTS

**Figure 1:**
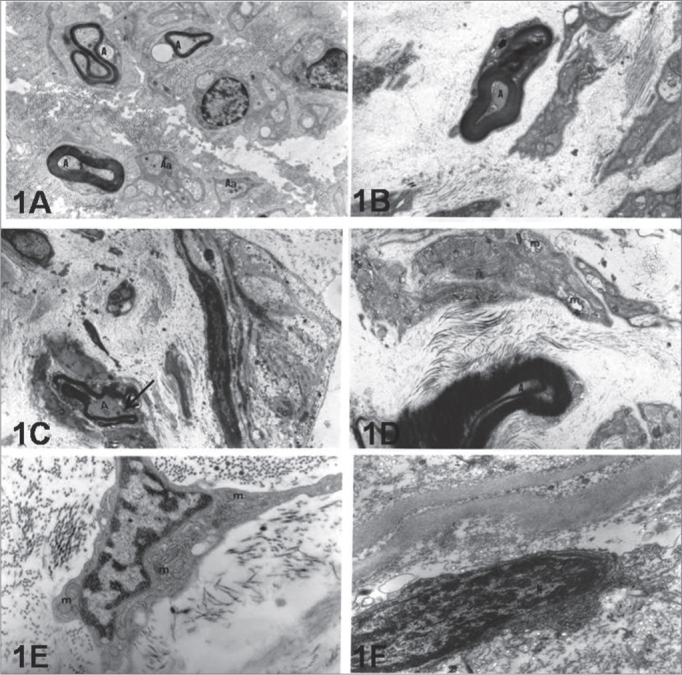
Electronic micro-photography showing parts of the intratemporal facial nerves of individuals with idiopathic facial paralysis (A, C and E) and without facial paralysis (B, D and F). Notice in 1A: myelinated axons (A) and amyelinated (Aa), in 1C myelin sheath break (Arrow) and, in 1E typical fibroblast indicating intense synthesis activity (m=mitochondria). In 1B we notice axon (A) coated by myelin lamellae in disarray (Arrow). In 1D: Schwann cell with mitochondria (m) with cristolysis signs. In 1F fibroblast with heterochromatic nucleus (N) indicating low synthesis activity. A, B and C = 12,000X; D, E and F = 24,000X.

The ultrastructural analysis done on biopsies of the facial nerves from patients with idiopathic PFP (GPFP.I) in comparison with the G-PFP.T group showed that in the majority of the fields read, the nerves were made up of myelinated and non-myelinated axons (Figs. 1A, B, C and D), which were coated by the endoneurium, perineurium and epineurium. Both the perineurium and the epineurium were mainly formed by type I collagen fibers. The axons were made up of typical mitochondria and neurofibrils.

In the PFP.I group, both the Schwann cells which coat the axons as the majority of extracellular matrix fibroblasts from the connective tissue showed ultrastructural characteristics of cells under intense synthesis activity, with volumous and euchromatic nuclei. In this group, the axons made feasible were coated by Schwann cells, and the myelinated fibers showed disorganizing lamellar pattern and basal lamina discontinuity (Fig. 1C). The fibroblasts showed cytoplasm with long extensions, rich in synthesis organelles, such as: granular endoplasmic reticulum and mitochondria (Figure 1E).

In preparations from patients from group PFP.T (trauma or tumoral origin) one of the findings was that of some axons with structural irregularities. In cross-section views these axons were flattened and coated by myelin lamellas in disarray (Fig. 1B). Now, some Schwann cells had cytoplasm with lysosomes and mitochondria with signs of cristolysis (Fig. 1D). Endoneurium, perineurium and epineurium are made up of fibroblasts with characteristics of low synthesis cells, showing reduced cytoplasm, heterochromatic nuclei and a few synthesis organelles (Fig. 1F). These cells are permeated by a large concentration of collagen fibrils, especially of the type I.

In none of the groups studied - STUDY and CONTROL - we identified the presence of viral particles.

## DISCUSSION

The histology study of facial nerves in facial paralysis started in 1869 with the use of light microscopy (LM) by Moxon, and a while later, in 1882, researchers noticed the degenerations of axons on the myelin sheath distally, starting on the geniculate ganglion, especially on the mastoid segment of the facial nerve, and also by using LM by Minkowski. Degenerations of these neural structures, seen under electron microscopy were confirmed in our study both in the control group as well as in the study group, and it was also shown by other authors^5^,^14^,^15^.

Comparing our results from the study group with literature reviews, we can make the following remarks: we've found fibroblasts in intense activity, edema and Schwann cells proliferation, as observed in a histology study of the facial nerve from a patient with PFP^4^. The authors suggest that the inflammatory changes and the edema found in this PFP case could be of viral etiology. However, in the normal process of facial nerve regeneration, there is also an increase in the number of cell organelles.

The study group had fibroblasts present in most of the histology cross-sections studied, with numerous organelles, showing us we had cells under intense synthesis activity. These cells were also surrounded by a large number of collagen fibers permeated by electro-translucent spaces, suggesting edema. These aspects are typical of a cell reacting to an inflammatory process.

In this comparative study, we looked for viral evidence on the nerves of patients with Bell's palsy (study group) and in the patients with tumoral or trauma facial paralysis (control group); however, we did not notice viral particles in any of the fragments.

In autopsy histology studies in idiopathic PFP, researchers found infiltrates of inflammatory cells in the nerve, myelin sheath destruction and important edema, which, according to the authors, suggested viral infection or even an immune reaction^10^. Our study presented results which were similar to the ones in the control group, in which we showed intense repair cell activity, with an increase in the number of active fibroblasts, thanks to the presence of developed organelles, which despite having no viral particles, could suggest viral infection, as previously reported.

In the control group this repair activity was not evident and viral particles were also not found. We found part of the nerve with normal neural structure, even in chronic paralysis, in which the individual had a complete functional neural lesion with unresponsive electromyography.

In our study we found a degeneration of myelin fibers in both groups, matching other studies which assessed the chorda tympani and petrous major nerves obtained during facial nerve decompression surgery^14^.

We must not forget that we also had technical difficulties in handling the facial nerve. To obtain proper facial nerve fragments for histology purposes during surgery is a hard and rare task. This is so because of the low number of these procedures which would allow for it, the artifact produced during the extraction process and specimen size, since the tissue fragment is usually very small. This turned the method very difficult, and such predicament has also been described by other authors^14^, ^15^, ^16^.

As to our failure in identifying viral particles, it was suggested that idiopathic PFP is a herpetic infection, in which the virus would be inside the ganglion^11^. During the proliferative phase, the ganglion's cell bodies are attacked and paralysis would ensue. Therefore, if the virus is inside the ganglion, we could not find it in fragments from the sheath, stumps or branches. This would be one more possibility to justify the absence of viral particles in our samples, adding to the other ones already discussed, such as the technical insufficiency of our method or even the occurrence of a viral etiology in Bell's PFP.

## CONCLUSIONS

We did not find evidence of viral infection (viral particles) in patients with idiopathic peripheral facial paralysis; nonetheless we found non-pathognomonic inflammatory evidence, suggesting viral infection.
